# Analysis of microbial composition and sharing in low-biomass human milk samples: a comparison of DNA isolation and sequencing techniques

**DOI:** 10.1038/s43705-023-00325-6

**Published:** 2023-11-09

**Authors:** Johanne E. Spreckels, Asier Fernández-Pato, Marloes Kruk, Alexander Kurilshikov, Sanzhima Garmaeva, Trishla Sinha, Hiren Ghosh, Hermie Harmsen, Jingyuan Fu, Ranko Gacesa, Alexandra Zhernakova

**Affiliations:** 1 Department of Genetics, University of Groningen and University Medical Center Groningen, Groningen, the Netherlands; 2 Medical Center - University of Freiburg, Institute for Infection Prevention and Hospital Epidemiology, Freiburg, Germany; 3 Department of Medical Microbiology, University of Groningen and University Medical Center Groningen, Groningen, the Netherlands; 4 Department of Pediatrics, University of Groningen and University Medical Center Groningen, Groningen, the Netherlands; 5 Department of Gastroenterology and Hepatology, University of Groningen and University Medical Center Groningen, Groningen, the Netherlands

**Keywords:** Microbiome, Sequencing

## Abstract

Human milk microbiome studies are currently hindered by low milk bacterial/human cell ratios and often rely on 16S rRNA gene sequencing, which limits downstream analyses. Here, we aimed to find a method to study milk bacteria and assess bacterial sharing between maternal and infant microbiota. We tested four DNA isolation methods, two bacterial enrichment methods and three sequencing methods on mock communities, milk samples and negative controls. Of the four DNA isolation kits, the DNeasy PowerSoil Pro (PS) and MagMAX Total Nucleic Acid Isolation (MX) kits provided consistent 16S rRNA gene sequencing results with low contamination. Neither enrichment method substantially decreased the human metagenomic sequencing read-depth. Long-read 16S-ITS-23S rRNA gene sequencing biased the mock community composition but provided consistent results for milk samples, with little contamination. In contrast to 16S rRNA gene sequencing, 16S-ITS-23S rRNA gene sequencing of milk, infant oral, infant faecal and maternal faecal DNA from 14 mother-infant pairs provided sufficient resolution to detect significantly more frequent sharing of bacteria between related pairs compared to unrelated pairs. In conclusion, PS or MX kit-DNA isolation followed by 16S rRNA gene sequencing reliably characterises human milk microbiota, and 16S-ITS-23S rRNA gene sequencing enables studies of bacterial transmission in low-biomass samples.

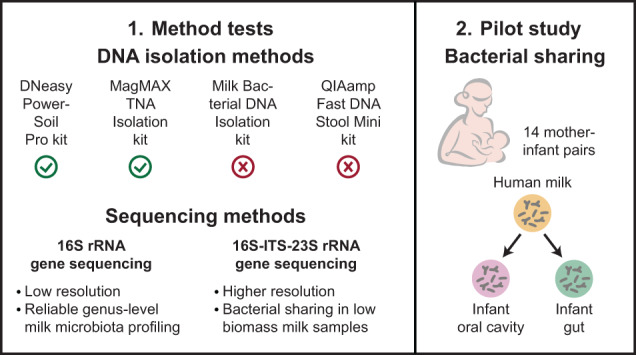

## Introduction

Breastfeeding provides infants with nutrients and is associated with infant health benefits [1, 2]. One way that breastfeeding can affect infant health is by modulating the gut microbiome [3] as human milk harbours bacteria and viruses that potentially originate from the maternal gut, skin and infant oral cavity [4–10]. Once ingested by the human milk-fed infant, milk microbes can colonise the developing infant gut [11–16], affecting the infant’s immune system maturation and health [3, 17, 18]. However, study of the origin of human milk microbiota and milk as a source of microbes to the early infant gut has been hindered by methodological challenges.

Human milk has a low microbial biomass, containing ~10^5^–10^6^ bacterial cells per 1 ml milk [9, 11]. It also contains many nutrients, which complicate isolation of milk DNA [12, 19–21]. Frequently, over 90% of DNA isolated from milk is of human rather than microbial origin [12, 19–21]. Most milk microbiota studies to date have therefore relied on 16S rRNA gene amplicon sequencing, which detects bacteria and archaea but limits taxonomic classification and the ability to study microbial transfer [7]. Metagenomic sequencing would resolve these limitations but remains methodologically challenging for milk samples due to their low-biomass and high host contamination [12, 19–21].

In this study, we aimed to identify a sample preparation and sequencing strategy that increases taxonomic resolution and allows the study of bacterial transmission in low-biomass milk samples. Of the four DNA isolation kits we tested, the DNeasy PowerSoil Pro (PS) kit and the MagMAX Total Nucleic Acid Isolation (MX) kit provided consistent results, with low contamination. We also found only limited enrichment when testing two bacterial enrichment methods prior to metagenomic sequencing. Finally, we show that long-read 16S-ITS-23S rRNA gene amplicon sequencing did not improve taxonomic classification compared to 16S rRNA gene sequencing. Still, when analysing samples of 14 mother-infant pairs, 16S-ITS-23S rRNA gene amplicon sequencing provided sufficient resolution to detect significantly more frequent sharing of bacteria between human milk and infant oral cavity and faeces, but not with maternal faeces, in related mother-infant pairs compared to unrelated pairs.

## Methods

### Samples

#### Method test samples

Human milk test samples were collected from three mothers participating in the Dutch Lifelines NEXT birth cohort [22] at 1.5 (Milk-1), 2 (Milk-2) and 6 (Milk-3) months postpartum. Milk-1 and Milk-3 were collected in three pumping sessions, with samples pooled and homogenised by pipetting up and down for 5 min. Aliquoted (pooled) milk was stored at −20 °C until use. As positive controls, we used two mock communities, the ZymoBiomics Microbial Community Standard (“bacterial mock community”, D6300, Zymo Research, Irvine, California, USA) and the ZymoBiomics Microbial Community DNA Standard (“DNA mock community”, D6305, Zymo Research), according to the manufacturer’s instructions (version 1.1.5 for D6300, version 1.1.6 for D6305). The expected composition of both mock communities (“theoretical mock”) is shown in Table S1. DEPC-treated water (46-2224, Invitrogen, Waltham, Massachusetts, USA) filtered through sterile 0.45 µm cellulose acetate filters (514-0063, VWR, Radnor, Pennsylvania, USA) was used for negative controls.

#### Pilot samples

Milk (*n* = 14), maternal faeces (*n* = 14), infant faeces (*n* = 14) and infant oral swabs (*n* = 14) were collected from 14 Lifelines NEXT mother-infant pairs at 3 months postpartum. Mothers were asked to collect milk, without specific cleaning of the breast tissue and using their usual breast pump, from one, preferably the right, breast from the second feeding after midnight and at least 2 h after the last feed from that breast. Mothers expressed a mean (range) of 90 (20–200) ml milk. They homogenised the milk by gentle shaking and aliquoted milk into cryotubes (122279, Greiner Bio-One, Kremsmünster, Austria) using plastic dropper pipettes (H10041, MLS, Menen, Belgium). Faecal samples were collected and aliquoted into cryotubes using plastic dropper pipettes. Milk and faecal samples were stored at −20 °C in home freezers. Infant oral samples were collected during a home visit by a research nurse who gently moved a cotton swab (479165, Paul Hartmann, Heidenheim, Germany) along the inside of the infant’s cheeks and under the infant’s tongue until the swab was fully wetted by saliva. The swabs were placed in cryotubes containing 500 µl PowerBead Solution (12955-4-BS, Qiagen, Venlo, Netherlands). Research nurses then transported all samples to the laboratory in transportable freezers and samples were stored at −20 °C (short-term) or −80 °C (long-term) until analysis. Mean (range) total storage time for milk was 17 (14–22) months and for other sample types 27 (24–30) months.

### DNA isolation of method test samples

DNA was isolated from method test samples using four DNA isolation kits: the PS kit (47014, Qiagen), the MX kit (AM1840, Thermo Fisher, Waltham, Massachusetts, USA), the Milk Bacterial DNA Isolation (MD) kit (21550, Norgen Biotek, Thorold, Canada) and the QIAamp Fast DNA Stool Mini (FS) kit (51604, Qiagen). DNA was isolated from milk and negative controls in triplicates (PS kit) or duplicates (MX, MD and FS kits) using 3.5 ml sample input. DNA from the bacterial mock community was isolated once per kit using 75 µl of the bacterial mock community sample mixed with 3.425 ml DEPC-treated water. Detailed isolation procedures are described below. All DNA eluates were stored at −20 °C until further processing.

#### DNeasy PowerSoil Pro kit

Samples were centrifuged at 13,000 × *g* at 4 °C for 15 min. Fat (for milk samples) and supernatants were removed. Pellets were resuspended in 800 µl Solution CD1, moved to PowerBead Pro tubes and vortexed briefly. To lyse cells, samples were incubated at 65 °C for 10 min and bead-beat at 5000 rpm at 4 °C for 45 s using a Precellys Evolution Homogenizer (Bertin Instruments, Rockville, Maryland, USA). Afterwards, samples were centrifuged at 15,000 × *g* at 4 °C for 1 min. Finally, DNA was automatically extracted from 600 µl supernatant using the ‘DNeasy PowerSoil Pro Kit with Inhibitor Removal Technology Protocol’ (version May 2018) with an elution volume of 50 µl on a Qiacube (Qiagen).

#### MagMAX Total Nucleic Acid Isolation kit

Bead Tubes were briefly centrifuged, and 235 µl Lysis/Binding Solution was added. Samples were centrifuged at 13 000 × *g* at 4 °C for 15 min. Fat (for milk samples) and supernatants were removed. Pellets were resuspended in 175 µl phosphate-buffered saline, and samples were transferred to Bead Tubes. Samples were bead-beat twice for 90 s at 6800 rpm at 4 °C using a Precellys Evolution Homogenizer, with a 5 min break between. DNA was then isolated manually according to the manufacturer’s instructions (version P/N 4385118 Revision C from July 2008), starting from protocol step B.II.3, and eluted in 30 µl Elution buffer.

#### Milk Bacterial DNA Isolation kit

Samples were centrifuged at 20,000 × *g* at 4 °C for 2 min. Fat (for milk samples) and supernatants were removed. Pellets were resuspended in 100 µl Resuspension Solution A with lysozyme and moved to fresh tubes. DNA was then isolated according to the manufacturer’s instructions (version PI21550-4 from 2015), starting from protocol step 1B.d, and DNA was eluted in 50 µl Elution Buffer B.

#### QIAamp Fast DNA Stool Mini kit

Samples were centrifuged at 13,000 × *g* at 4 °C for 15 min. Fat (for milk samples) and supernatants were removed. The InhibitEX buffer was heated to 42 °C in a water bath for 10–15 min until crystals were dissolved. Pellets were then resuspended in 500 µl InhibitEX buffer and vortexed at maximum speed for 1 min. Samples were moved to fresh tubes, incubated at 95 °C for 10 min on a heating block (5355, Thermomixer Comfort, Eppendorf, Hamburg, Germany) shaking at 1000 rpm and then vortexed at maximum speed for 15 s. Samples were then centrifuged at 16,300 × *g* for 1 min, and 200 µl supernatant was used for automatic DNA extraction on a Qiacube using the ‘QIAamp Fast DNA Stool Mini Protocol for Isolation of DNA from Stool for Pathogen Detection’ (version March 2014) with an elution volume of 100 µl.

### DNA isolation of pilot samples

DNA was isolated once from each pilot sample using the PS kit. For milk samples, DNA was isolated from 3.5 ml milk, as described above. For faeces and oral swabs, we adapted the isolation procedure prior to the heat incubation and bacterial lysis steps described above. Approximately 0.2–0.5 g faeces and 800 µl Solution CD1 were added to PowerBead Pro tubes and vortexed briefly. Oral swabs in PowerBead solution were vigorously vortexed for 3 min, swabs were removed and ~400 µl solution per sample was transferred to PowerBead tubes. Solution CD1 was added to obtain a final volume of 800 µl, and samples were briefly vortexed before continuing the protocol from the heat incubation step. DNA was stored at −20 °C until further processing.

### DNA quantification

To measure DNA concentrations, the Qubit 1X dsDNA HS Assay Kit (Q33230, Invitrogen) was used according to the manufacturer’s instructions (version MAN0017455 Rev. C.0).

### Quantitative PCR

To quantify bacteria, we used quantitative PCR (qPCR) of the 16S rRNA gene as described in the Supplementary Methods.

### 16S rRNA gene sequencing and data processing

DNA was used for library preparation and 16S rRNA gene sequencing at the Institute of Clinical Molecular Biology at the Christian-Albrecht University of Kiel, Germany. The V3–V4 region of the bacterial 16S rRNA gene was amplified using 3 µl DNA and the 357F (5′-CCTACGGGAGGCAGCAG-3′) and 806R (5′-GGACTACHVGGGTWTCTAAT-3′) primer pair according to the dual-barcoding approach from Kozich et al. [23]. PCR products were checked using gel electrophoresis, normalised using the SequalPrep Normalisation Plate Kit (A1051001, Thermo Fisher) and pooled using 1.85 ng PCR product per sample. Pooled libraries were sequenced on Illumina MiSeq v3 systems (2 × 300 bp paired-end sequencing, Illumina, San Diego, California, USA).

After sequencing, samples were demultiplexed based on zero mismatches in barcode sequences. Adapters and primers were removed from sequencing reads using Cutadapt (version 2.6). Reads were quality-trimmed with Trimmomatic (version 0.39, parameters: paired-end mode, SLIDINGWINDOW:4:25 and MINLEN:50). Read quality was investigated with FastQC (version 0.11.5) and MultiQC (version 1.7). A modified version of the DADA2 pipeline (version 1.16, https://benjjneb.github.io/dada2/tutorial.html) was used to construct amplicon sequence variant (ASV) tables. Briefly, trimmed reads with at least 160 bp length were used to learn error rates, denoise and generate ASV tables. Chimaeras were removed, and only ASVs with 400–431 bp length were kept. Taxonomies were assigned to ASVs using the SILVA (version 138.1) and FANGORN databases (GTDB Full 207 database, https://melbourne.figshare.com/articles/dataset/Fangorn_rrn_Database/20086916 [24]). ASVs assigned as chloroplasts and mitochondria were removed from the SILVA dataset. Relative abundances were calculated for both the SILVA and FANGORN datasets.

### 16S-ITS-23S rRNA gene sequencing and data processing

DNA was prepared for 16S-ITS-23S rRNA gene sequencing using the Shoreline Wave^TM^ StrainID^TM^ kit (WAVESID-A, Shoreline Biome, Farmington, Connecticut, USA) according to the manufacturer’s instructions (version USM-050-0). Library preparation and sequencing were performed by BaseClear (Leiden, Netherlands). Briefly, samples were marked with unique barcodes, and the ~2500 bp-long 16S-ITS-23S rRNA gene fragment was amplified in a single-step PCR. Amplicons were pooled (5 µl PCR product per sample), used for SMRTbell® library preparation (100-938-900, PacBio, Menlo Park, California, USA) and sequenced on one Sequel II SMRT Cell on a PacBio Sequel System (PacBio), generating circular consensus reads.

Samples were demultiplexed and primers were removed from sequencing reads using SBanalyzer software (version 3.1-2, Shoreline Biome). The DADA2 pipeline was adapted for 16S-ITS-23S rRNA gene sequencing data. Reads were filtered and trimmed with modified settings (minQ=2, maxEE=5, trimLeft=20, trimRight=0, minLen=1900, maxLen=3000), dereplicated and used to learn error rates, denoise and construct the ASV table. Chimaeras were removed. Taxonomies were assigned using the FANGORN database [24], and relative abundances were calculated.

### Bacterial enrichment and shotgun metagenomic sequencing

We used two bacterial enrichment methods, hypotonic lysis and benzonase treatment (HL-Benz) [25] and hypotonic lysis and propidium monoazide treatment (HL-PMA) [26] on the method test samples, followed by DNA isolation and shotgun metagenomic sequencing. A detailed description for this can be found in the Supplementary Methods.

### Statistical analyses

We compared categorical and continuous parameters using Fisher’s exact tests and Mann–Whitney *U* tests, respectively. To compare continuous parameters between more than two categories, we used Kruskal–Wallis tests with Dunn’s post hoc tests. Spearman’s rank correlation was used to investigate correlation between continuous variables.

Relative bacterial abundances were centred log-ratio transformed and compared between methods using linear regression models with correction for milk sample as a fixed effect. We used the *vegdist* function from R package *vegan* to calculate Aitchison distances between samples based on the relative abundances of classified genera or (un)classified species. We used these distances to cluster mock communities or milk and negative control samples and to compare bacterial composition between samples using ‘Permutational Multivariate Analyses of Variances Using Distance Matrices’ with the *adonis* function.

To explore sharing of bacterial ASVs between human milk and maternal and infant samples, we selected ASVs belonging to genera present in at least two related sample pairs and aligned them with multiple sequence alignment (MSA) using R package *msa* with the ClustalW method. Based on the MSA, we generated a Hamming distance matrix for the ASVs of each genus using snp-dists (version 0.7.0, https://github.com/tseemann/snp-dists). This matrix was used to detect potential ASV-sharing events (presence of identical ASVs in a sample pair), and their frequency was compared between related and unrelated sample pairs using Fisher’s exact tests. In addition, we generated phylogenetic trees for *Streptococcus* ASVs using RAxML-NG (version 1.3, https://github.com/amkozlov/raxml-ng) with the GTR+G model and 1000 bootstrap replicates.

All statistical analyses were performed and all plots were created in RStudio (version 1.1.463) or R (version 4.2.2) using the R packages *ape* [27], *cowplot* [28], *dplyr* [29], *FSA* [30], *ggplot2* [31], *ggdendro* [32], *ggpubr* [33], *msa* [34], *plyr* [35], *reshape* [36], *phangorn* [37, 38], *seqinr* [39], *stringr* [40], *tidyr* [41] and *vegan* [42]. *P* values were adjusted using the Benjamini–Hochberg method. A false discovery rate (FDR) < 0.05 was considered statistically significant.

## Results

### Study design

We tested four DNA isolation methods, two bacterial enrichment methods and three sequencing methods on test samples: bacterial and DNA mock communities, a negative control and three human milk samples. We then applied one of the best-performing DNA isolation methods (the PS kit) and two sequencing methods (16S and 16S-ITS-23S rRNA gene sequencing) to samples from 14 mother-infant pairs to investigate their ability to detect bacterial sharing between human milk, maternal and infant faeces and infant oral swabs.

### Comparison of DNA isolation methods

**Fig. 1:**
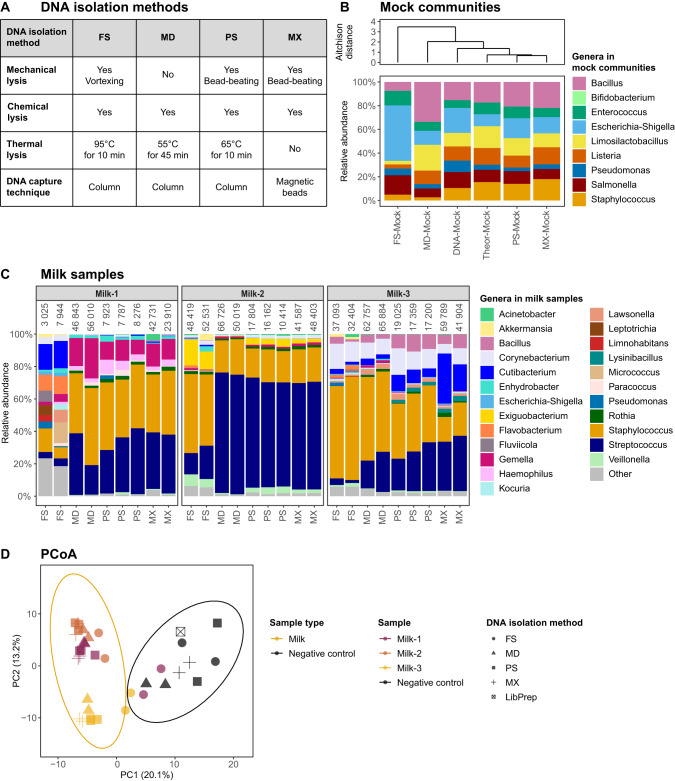
Comparison of DNA isolation methods. DNA was isolated from test samples using four DNA isolation kits, and samples were sequenced using 16S rRNA gene sequencing. Bacterial taxonomies were assigned using the SILVA database and all results are based on classified genera. **A** Information about DNA isolation protocols. **B** Relative bacterial abundances of mock communities, including bacterial mock communities isolated with the four DNA isolation kits, a DNA mock community (DNA-Mock, added at the start of library preparation) and a theoretical mock community (Theor-Mock, see Table S1). Mock communities are clustered based on Aitchison distances. **C** Relative bacterial abundances of milk samples isolated with the four DNA isolation kits. Numbers above the bars indicate the number of sequencing reads of the respective sample. Only classified bacterial genera present in at least two samples with at least 2% relative abundance in any sample are shown. Genera present at lower prevalence and abundance or without genus-level classification are aggregated in ‘Other’. **D** Principal Coordinates Analysis (PCoA) plot of the Aitchison distance matrix for milk samples and negative controls. The percentage of variance explained by the PCoAs is shown in the axis’s labels. Dot colour indicates the sample (type). Dot shape indicates DNA isolation method. Ellipses represent 95% confidence intervals. Sample type, sample (donor) and DNA isolation method significantly affected the bacterial composition, including when comparing only milk samples and excluding negative controls (Adonis, FDR < 0.05). FS QIAamp Fast DNA Stool Mini kit, MD Milk Bacterial DNA Isolation kit, PS DNeasy PowerSoil Pro kit, MX MagMAX Total Nucleic Acid Isolation kit, LibPrep Library preparation.

To find an efficient, reliable method for milk DNA isolation, we tested the FS, MD, PS and MX kits on method test samples (Fig. 1A). DNA isolation was followed by 16S rRNA gene sequencing and taxonomic classification with the SILVA database.

#### Mock communities and negative controls

The DNA yield from the bacterial mock community was highest with the MD kit, followed by the PS, MX and FS kits (Table 1A). The highest sequencing read count was obtained with the FS kit, followed by the MD, MX and PS kits (Table 1A). We compared the bacterial composition of isolated bacterial mock communities to both the theoretical mock composition (Table S1) and to results for a DNA mock community. All four DNA isolation methods detected all eight bacterial genera expected in the mock community, but their relative abundances varied between methods (Fig. 1B). The composition of the bacterial mock communities isolated with the MX and PS kits were closest to the theoretical composition (Fig. 1B), whereas the mock communities isolated with the MD and FS kits clustered further apart and showed increases in *Bacillus* and *Limosilactobacillus* (MD kit) or *Escherichia-Shigella* (FS kit) and decreases in *Staphylococcus* (both kits). Species-level characterisation of bacteria was only possible for 0.5–3.4% of the mock sequencing reads (Table 1A, Fig. S1). In the bacterial mock community isolated with the FS kit, we unexpectedly detected traces of *Bifidobacterium* (<0.02% relative abundance).

**Table 1:** DNA yield and sequencing parameters for mock communities (A), negative controls (B), and milk samples (C), isolated with four different DNA isolation methods.

A: Mock communities					
DNA isolation method	FS kit	MD kit	PS kit	MX kit	LibPrep
**Sample**	Bacterial mock community	Bacterial mock community	Bacterial mock community	Bacterial mock community	DNA mock community
*n* (samples)	1	1	1	1	1
DNA yield (ng)	3.5	367	307	138	NA
*n* (reads)	46454	23423	17044	22235	23789
*n* (ASVs)	14	12	12	15	12
*n* (classified genera)	9	8	8	8	8
% (reads classified on species level)	3.3%	0.5%	2.2%	3.4%	1.5%

B: Negative controls					
DNA isolation method	FS kit	MD kit	PS kit	MX kit	LibPrep
**Samples**	DNA isolation negative controls	DNA isolation negative controls	DNA isolation negative controls	DNA isolation negative controls	Library preparation negative control
*n* (samples)	2	2	3	2	1
DNA yield (ng)	0 (0–0)^a^	0 (0–0)^a^	0 (0–0)^a^	0 (0–0)^a^	NA
*n* (reads)	3726 (2899–4553)	4109 (3327–4890)	675 (0–808)	133 (127–138)	129 (129–129)
*n* (ASVs)	26 (26–26)	20 (19–20)	11 (9–13)^b^	5 (4–5)	4 (4–4)
*n* (classified genera)	14 (12–15)	17 (15–18)	11 (9–13)^b^	4 (4–4)	4 (4–4)

C: Milk samples						
DNA isolation method	FS kit	MD kit	PS kit	MX kit	p	FDR
**Samples**	3 milk samples in duplicate	3 milk samples in duplicate	3 milk samples in triplicate	3 milk samples in duplicate		
*n* (samples)	6	6	9	6		
DNA yield (ng)	8.0 (3.4–17.3)	34 (6.2–67)	240 (103–725)	227 (80–288)	0.0002	0.001
*n* (reads)	34749 (3025–52531)	59384 (46843–66726)	16162 (7787–19025)	42318 (23910–59789)	0.001	0.002
*n* (ASVs)	45 (28–60)	42 (35–60)	34 (21–52)	53 (42–63)	0.02	0.04
*n* (classified genera)	22 (20–29)	20 (16–31)	16 (13–26)	24 (20–38)	0.09	0.10
*n* (classified genera unique to milk)	17 (14–24)	16 (12–27)	14 (12–24)	23 (19–37)	0.09	0.10
% (reads shared with negative controls)	9% (2%–39%)	6% (1%–7%)	3% (0%–10%)	1% (0%–29%)	0.10	0.10
*n* (16S rRNA gene copies per 1 ml milk)	NA	NA	10^5.0^ (10^4.8^–10^5.2^)^c^	NA	NA	NA

DNA was isolated from test samples and sequenced with 16S rRNA gene sequencing (V3–V4 region). Bacterial taxonomies were assigned using the SILVA database. For *(B) and (C)*, values represent median (range). Parameters for milk samples in *(C)* were compared using Kruskal–Wallis tests with Benjamini–Hochberg correction for multiple testing.

*FS* QIAamp Fast DNA Stool Mini Kit, *MD* Milk Bacterial DNA Isolation Kit, *PS* DNeasy PowerSoil Pro Kit, *MX* MagMAX Total Nucleic Acid Isolation Kit, *LibPrep* Library preparation, *NA* not applicable.

^a^DNA concentrations were below the detection limit of the method and the DNA yield was set to 0.

^b^One negative control had 0 reads and was excluded for these parameters.

^c^16S rRNA gene copy numbers per 1 ml milk used for DNA isolation were only determined for the first two replicates of each of the three PS kit-isolated milk samples. For 1 of the Milk-1 samples, no 16S rRNA gene copies could be measured and this sample was excluded for this parameter.

All negative controls had DNA concentrations below the detection limit of the method and low read counts (median (range): 742 (0–4 890) reads) (Table 1B). Negative controls isolated with the MX kit had the lowest median number of reads, followed by the PS, FS and MD kits (Table 1B). *Cutibacterium*, *Enhydrobacter*, *Escherichia-Shigella* and *Pelomonas* were commonly detected in isolation-negative controls (Fig. S2, Table S2).

#### Milk samples

The median DNA yield from milk samples was significantly higher with the PS and MX kits compared to the MD and FS kits (FDR=0.001, Table 1C). The MD kit resulted in significantly higher read counts than the FS and PS kits, while the MX kit led to significantly higher read counts than the PS kit (FDR < 0.05, Table 1C). The median number of bacterial genera detected in milk was similar between the isolation methods (Table 1C).

Nine bacterial genera were present in at least 70% of milk samples: *Acinetobacter*, *Corynebacterium*, *Cutibacterium*, *Enhydrobacter*, *Gemella*, *Rothia*, *Staphylococcus*, *Streptococcus* and *Veillonella* (Fig. 1C, Table S3). The most abundant bacterial genera detected in milk samples (≥15% relative abundance in ≥1 milk sample) were *Staphylococcus* (all kits), *Streptococcus* (all kits), *Cutibacterium* (FS and MX kits), *Corynebacterium* (FS and PS kits), *Gemella* (MD kit) and *Exiguobacterium* (FS kit) (Fig. 1C).

The overall milk bacterial composition (beta diversity) differed significantly between mothers (*R*^2^ = 0.37, FDR = 0.002, Fig. 1D). The DNA isolation method also significantly affected milk beta diversity (*R*^2^ = 0.19, FDR = 0.01, Fig. 1D), and the relative abundances of five of the nine most-prevalent genera in milk differed significantly between the DNA isolation methods (*Corynebacterium*, *Cutibacterium*, *Rothia*, *Staphylococcus* and *Streptococcus*, all FDR < 0.05, Table S3). Importantly, the bacterial composition of milk differed significantly from that of negative controls (*R*^2^ = 0.17, FDR = 0.002, Fig. 1D). In addition, the median number of sequencing reads for all negative controls (742 reads) was significantly lower than for all milk samples (37,093 reads, *p* < 0.001). Bacteria detected in milk and negative controls are shown in Table S4. *Cutibacterium* was detected in milk and corresponding negative controls by all four kits and *Enhydrobacter* by three kits (Table S4).

Next, we quantified the extent of potential contamination in milk sample results. An ASV detected in both a milk sample and a corresponding negative control was considered a potential contaminant. When comparing DNA isolation methods, we saw no significant difference in the percentage of sequencing reads that contained a potentially contaminating ASV (Table 1C). However, the percentage of reads corresponding to ASVs in negative controls varied the most for milk samples isolated with the FS and MX kits and the least for milk samples isolated with the MD and PS kits (Table 1C). By comparing the negative controls from isolation and library preparation, we concluded that contamination was frequently introduced during DNA isolation (range 0.1–39.1% across DNA isolation methods) but very rare during library preparation (range 0–0.7%) (Table S4). There was also a significant negative correlation between DNA yield and the percentage of contamination (Spearman’s rho = −0.56, FDR = 0.005) but not between the number of milk sequencing reads and the percentage of contamination (FDR = 0.98).

Based on these analyses, we concluded that the PS and MX kits were the most suitable for milk DNA isolation because they represented the mock community well, led to little contamination and produced similar 16S rRNA gene sequencing results for milk sample replicates. We decided to continue our following tests with the PS kit for three reasons: (1) the maximum percentage of sequencing reads shared with negative controls was smaller for the PS than for the MX kit, (2) we had DNA isolation robots for the PS kit available in our lab and (3) we experienced difficulties with separating magnetic beads from milk DNA eluates in the elution step of the MX kit. Before continuing with the next tests, we further showed that the PS kit detected a median of 10^5^ 16S rRNA gene copy numbers per 1 ml human milk (Table 1C), which was similar to other studies [9, 11].

### Bacterial enrichment and metagenomic sequencing

We next aimed to enrich milk bacterial DNA prior to metagenomic sequencing. Here we tried two bacterial enrichment methods, HL-Benz and HL-PMA, combined with metagenomic sequencing on the test samples (Table S5). Library preparation or sequencing was not successful for any samples prepared with the HL-Benz method and was only successful for four of nine milk samples and the bacterial mock community prepared with the HL-PMA method. For the nine non-enriched and five HL-PMA-enriched milk samples that could be sequenced, at least 98.8% of the sequencing reads were of human origin and only a median of <1% was of microbial origin (Table S5). We therefore concluded that these bacterial enrichment methods did not substantially decrease the proportion of human reads and thus they were not efficient for use with human milk samples in combination with metagenomic sequencing.

### Comparison of 16S and 16S-ITS-23S rRNA gene sequencing

**Fig. 2:**
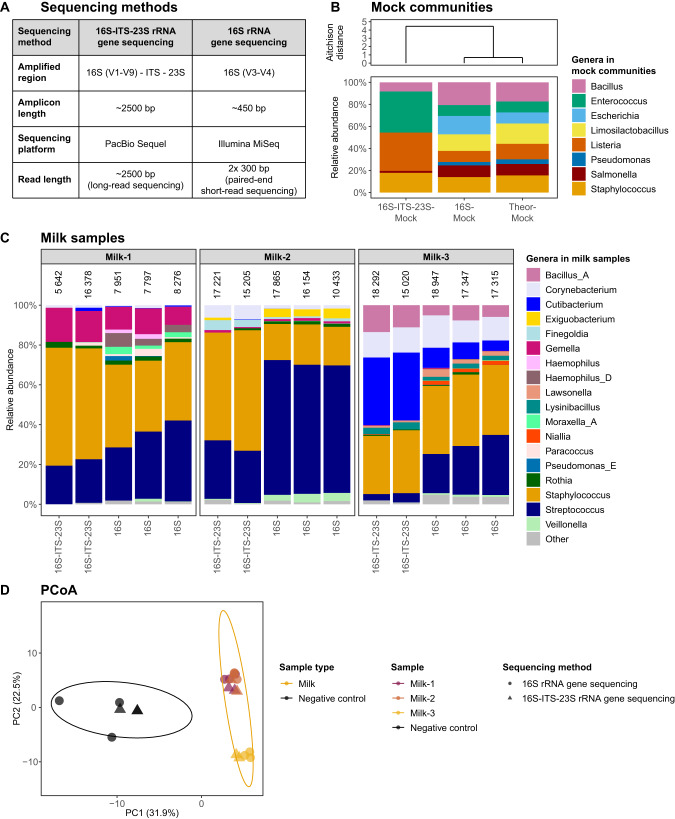
Comparison of sequencing methods. DNA was isolated from test samples using the DNeasy PowerSoil Pro kit, and samples were sequenced with 16S and 16S-ITS-23S rRNA gene sequencing. Bacterial taxonomies were assigned using the FANGORN database, and all results are based on classified genera. **A** Information about the sequencing methods. **B** Relative bacterial abundances of mock communities, including isolated bacterial mock community DNA sequenced with each sequencing method and a theoretical mock community (Theor-Mock, see Table S1). Mock communities are clustered based on Aitchison distances. **C** Relative bacterial abundances of milk samples obtained by 16S and 16S-ITS-23S rRNA gene sequencing. Numbers above the bars indicate the number of sequencing reads of the respective sample. Only classified bacterial genera present in at least two samples with at least 2% relative abundance in any sample are shown. Genera present at lower prevalence and abundance or without genus-level classification are aggregated in ‘Other’. **D** Principal Coordinates Analysis (PCoA) plot of the Aitchison distance matrix for milk samples and negative controls. The percentage of variance explained by the PCoAs is shown in the axis’s labels. Dot colour indicates the sample (type). Dot shape indicates sequencing method. Ellipses represent 95% confidence intervals. Sample type and sample (donor) significantly affected the bacterial composition of milk and negative controls, including when comparing only milk samples and excluding negative controls (FDR = 0.02). Sequencing method did not significantly affect the bacterial composition.

We next aimed to improve taxonomic resolution of milk microbiota profiling. For this, we isolated test sample DNA with the PS kit and subjected it to both 16S-ITS-23S and 16S rRNA gene sequencing (Fig. 2A). To compare results between the sequencing methods, all bacteria were classified using the FANGORN database, which contains full-length 16S-ITS-23S rRNA operon sequences [24].

#### Mock communities and negative controls

16S rRNA gene sequencing identified all eight genera in the mock community, and the bacterial composition closely resembled the theoretical composition (Table 2A, Fig. 2B). When using 16S-ITS-23S rRNA gene sequencing, only five of the eight genera of the mock community were detected (*Bacillus*, *Enterococcus*, *Listeria*, *Salmonella* and *Staphylococcus*), while *Escherichia*, *Limosilactobacillus* and *Pseudomonas* were missing (Table 2A, Fig. 2B). Species-level characterisation of bacteria was improved with the FANGORN database, though *Bacillus subtilis* and *Escherichia coli* (if detected) were misclassified, making species-level classification unreliable (Fig. S3). Negative controls sequenced with 16S-ITS-23S rRNA gene sequencing had a maximum of seven reads (Table 2B, Table S6).

**Table 2:** Sequencing parameters for mock communities (A), negative controls (B), and milk samples (C), sequenced with 16S and 16S-ITS-23S rRNA gene sequencing.

A: Mock communities		
Sequencing method	16S-ITS-23S rRNA gene sequencing	16S rRNA gene sequencing
**Sample**	Bacterial mock community	Bacterial mock community
*n* (samples)	1	1
*n* (reads)	3336	17123
*n* (ASVs)	11	12
*n* (classified genera)	5	8

B: Negative controls				
Sequencing method	16S-ITS-23S rRNA gene sequencing	16S-ITS-23S rRNA gene sequencing	16S rRNA gene sequencing	16S rRNA gene sequencing
**Samples**	DNA isolation negative controls	Library preparation negative control	DNA isolation negative controls	Library preparation negative control
*n* (samples)	2	1	3	1
*n* (reads)	4 (1–7)	1 (1–1)	675 (0–808)	129 (129–129)
*n* (ASVs)	1 (1–1)	1 (1–1)	11 (9–13)	4 (4–4)
*n* (classified genera)	1 (0–1)	0 (0–0)	9 (8–9)	3 (3–3)

C: Milk samples				
Sequencing method	16S-ITS-23S rRNA gene sequencing	16S rRNA gene sequencing	p	FDR
**Samples**	3 milk samples in duplicate	3 milk samples in triplicate		
*n* (samples)	6	9		
*n* (reads)	15792 (5642–18292)	16154 (7797–18947)	0.95	0.96
*n* (ASVs)	38 (25–45)	35 (21–47)	0.81	0.96
*n* (classified genera)	12 (5–15)	16 (11–28)	0.02	0.046

DNA was isolated from test samples using the PowerSoil Pro DNA isolation kit. Samples were sequenced using 16S rRNA gene sequencing (V3–V4 region) and long-read 16S-ITS-23S rRNA gene sequencing. Bacterial taxonomies were assigned using the FANGORN database. Values represent median (range). Parameters for milk samples in *(C)* were compared using Mann–Whitney *U* tests and *p* values were corrected for multiple testing using the Benjamini–Hochberg correction.

#### Milk samples

The number of sequencing reads obtained for milk samples did not differ between 16S-ITS-23S and 16S rRNA gene sequencing (Table 2C). On average, four more genera were detected in milk with 16S than with 16S-ITS-23S rRNA gene sequencing (FDR=0.046, Table 2C). Seven bacterial genera (*Corynebacterium*, *Cutibacterium*, *Gemella*, *Rothia*, *Staphylococcus*, *Streptococcus* and *Veillonella*) were present in at least 70% of the milk samples profiled by both 16S and 16S-ITS-23S rRNA gene sequencing (Table S7). The most abundant genera (≥15% relative abundance in ≥1 milk sample) were *Staphylococcus* and *Streptococcus* (both methods), *Cutibacterium* and *Gemella* (16S-ITS-23S rRNA gene sequencing) and *Corynebacterium* (16S rRNA gene sequencing) (Fig. 2C). The overall bacterial composition of milk samples differed significantly between mothers (*R*^2^ = 0.63, FDR=0.002) and from that of negative controls (R^2^=0.30, FDR=0.002, Fig. 2D). The sequencing method had no significant effect on milk beta diversity (FDR=0.21, Fig. 2D). Higher relative abundances of *Corynebacterium* and *Staphylococcus* and lower relative abundances of *Streptococcus* and *Veillonella* were detected with 16S-ITS-23S compared to 16S rRNA gene sequencing (all FDR=0.01, Table S7).

#### Bacterial sharing in mother-infant pairs

**Fig. 3:**
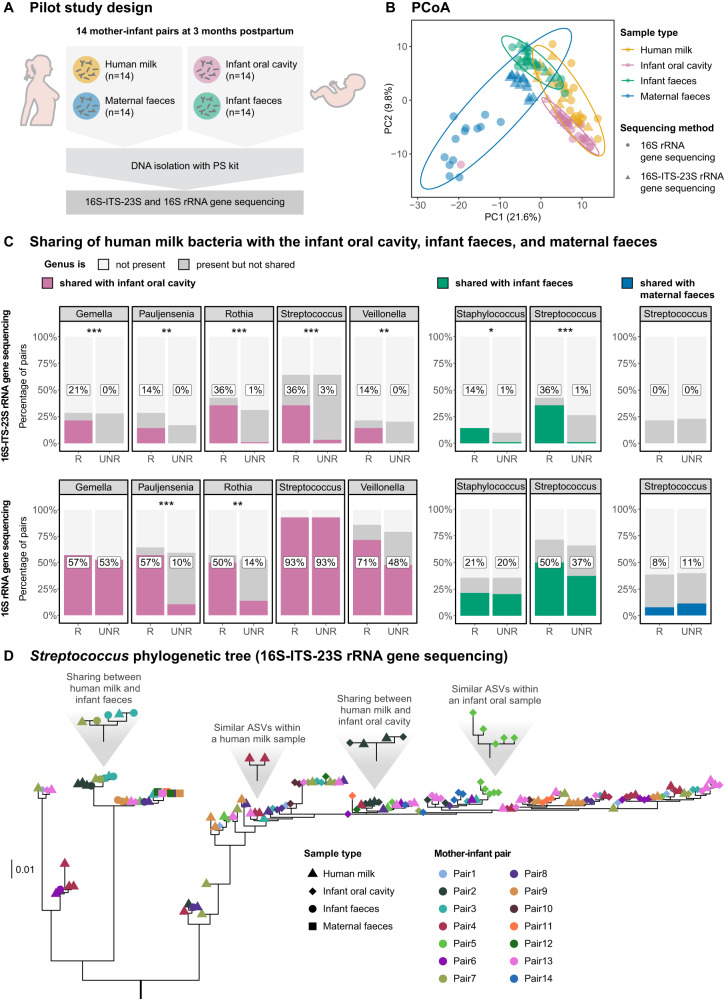
Milk bacterial sharing with maternal and infant body sites by sequencing method. **A** Study design. DNA was isolated from human milk, infant oral swabs and maternal and infant faeces using the DNeasy PowerSoil Pro (PS) kit. Samples were sequenced with 16S and 16S-ITS-23S rRNA gene sequencing, and bacterial taxonomies were assigned using the FANGORN database. One maternal faecal sample failed 16S rRNA gene sequencing. **B** Principal Coordinates Analysis (PCoA) plot of the Aitchison distance matrix for classified bacterial genera in different maternal and infant samples. The percentage of variance explained by the PCoAs is shown in the axis’s labels. Dot colour indicates sample type. Dot shape indicates sequencing method. Ellipses represent 95% confidence intervals. Both the sample type and the sequencing method significantly affected the bacterial composition (FDR=0.002). **C** Sharing of bacterial ASVs between human milk and infant oral cavity, infant faeces and maternal faeces, per sequencing method (top row=16S-ITS-23S rRNA gene sequencing, bottom row=16S rRNA gene sequencing). Bars show the percentage of related (R) and unrelated (UNR) sample pairs in which the indicated genus is not present in both sample types (light grey), present but not shared in both sample types (dark grey) or shared between both sample types (coloured). Percentages in the plot indicate the percentage of pairs with sharing. Only genera present in at least two sample pairs with both sequencing methods are shown. Comparisons include 14 related pairs and 182 unrelated pairs, except for linking human milk and maternal faecal data from 16S rRNA gene sequencing, which included 13 related and 169 unrelated pairs. Statistically significant results (Fisher’s exact test): *FDR<0.05, **FDR<0.01, ***FDR<0.001. **D** Phylogenetic tree for *Streptococcus* based on 16S-ITS-23S rRNA gene sequencing data. Dot shape indicates sample type. Dot colour indicates the mother-infant pair ID. Phylogenetic distances were log-transformed for plotting. A phylogenetic tree for *Streptococcus* based on 16S rRNA gene sequencing data is shown in Fig. S8.

Finally, we investigated the ability of 16S and 16S-ITS-23S rRNA gene sequencing to detect sharing of bacterial ASVs between human milk and infant oral cavity, infant faeces and maternal faeces (Fig. 3A). The total bacterial load (16S rRNA gene copy numbers per 1 ng DNA) differed significantly between sample types, with human milk having the lowest and maternal faeces having the highest number of 16S rRNA gene copies (Fig. S4). Also, the overall bacterial composition differed significantly between sample types (*R*^2^ = 0.27, FDR=0.002) and sequencing methods (*R*^2^ = 0.03, FDR=0.002, Fig. 3B, Fig. S5). When investigating the effect of the sequencing methods separately for each sample type, the method used significantly affected the infant oral (*R*^2^ = 0.15, FDR=0.002) and maternal faecal bacterial composition (*R*^2^ = 0.28, FDR=0.002) but not the milk (FDR=0.16) and infant faecal bacterial composition (FDR=0.59). 16S rRNA gene sequencing identified significantly more genera in each sample type than 16S-ITS-23S rRNA gene sequencing (Fig. S6). Interestingly, the bacterial genera missing in the mock community sequenced with 16S-ITS-23S rRNA gene sequencing (Fig. 2B), *Escherichia*, *Limosilactobacillus* and *Pseudomonas*, were detected by 16S-ITS-23S rRNA gene sequencing in some milk and faecal samples (Fig. S5, Table S8).

The number of genera detected in both human milk and another sample type from related mother-infant pairs (potentially shared genera) was consistently higher when using 16S than when using 16S-ITS-23S rRNA gene sequencing (21 vs. 10 genera for human milk-infant oral cavity, 14 vs. 5 genera for human milk-infant faeces, 5 genera vs. 1 genus for human milk-maternal faeces, Fig. S7). We focused on investigating bacterial sharing based on exact ASV-matching for potentially shared genera detected in at least two sample pairs with both sequencing methods (Fig. 3C, Fig. S7). For these potentially shared genera, the percentage of unrelated sample pairs that shared at least one ASV of the investigated genus was significantly higher with 16S (range 10–93%) than with 16S-ITS-23S rRNA gene sequencing (range 0–3%, all FDR<0.05, Fig. 3C, Table S9). With 16S-ITS-23S rRNA gene sequencing, significantly more related human milk-infant oral swab sample pairs shared ASVs belonging to *Gemella*, *Pauljensenia*, *Rothia*, *Streptococcus* and *Veillonella* genera when compared to unrelated pairs (Fig. 3C). 16S rRNA gene sequencing also detected significantly more sharing of *Pauljensenia* and *Rothia*, but not of *Gemella*, *Streptococcus* and *Veillonella*, in related human milk-infant oral swab pairs compared to unrelated pairs (Fig. 3C).

When comparing human milk and infant faeces, 16S-ITS-23S rRNA gene sequencing detected significantly more sharing of *Staphylococcus* and *Streptococcus* between related pairs compared to unrelated pairs, whereas 16S rRNA gene sequencing did not (Fig. 3C). 16S-ITS-23S rRNA gene sequencing detected no bacterial sharing between maternal faeces and human milk (Fig. 3C). By contrast, 16S rRNA gene sequencing found sharing of *Streptococcus*, although there was no significant difference between related and unrelated pairs (Fig. 3C). As *Streptococcus* was identified as potentially shared across all four sample types, we constructed phylogenetic trees for *Streptococcus* based on both 16S and 16S-ITS-23S rRNA gene sequencing data (Fig. 3D, Fig. S8). While the resolution of 16S rRNA gene sequencing data was too low to separate bacterial strains, hindering the study of within-sample and within-family clustering (Fig. S8), 16S-ITS-23S rRNA gene sequencing provided higher phylogenetic resolution and richer ramification, which revealed higher ASV similarity within individuals and families compared to unrelated individuals (Fig. 3D).

In summary, 16S-ITS-23S rRNA gene sequencing did not provide a complete representation of bacterial communities or reliable species-level classifications, but it was much less affected by potential contamination. 16S-ITS-23S rRNA gene sequencing was able to detect sharing of human milk bacteria with the infant’s oral cavity and faeces, but not with the maternal faeces, in more related pairs than unrelated mother-infant pairs.

## Discussion

### DNA isolation methods affect microbiota results

The DNA isolation methods we tested, or previous versions of them, have been used to isolate DNA for milk microbiota studies by other groups (FS kit [43–47], MD kit [48], PS kit [15, 21, 49]) or in our lab (MX kit). A good DNA isolation method should lyse all the bacteria in a sample, introduce minimal bias to the relative community composition, provide sufficient DNA for downstream analyses and not introduce contaminants. Previous studies describe that DNA isolation methods affect microbiota results and that mechanical lysis is important for efficient DNA isolation and accurate profiling of gram-positive bacteria [50–54]. In concordance with this, our 16S rRNA gene sequencing results differed between DNA isolation methods. Differences in isolation procedures, such as different sample centrifugation settings, lysis methods, and DNA capturing techniques, likely influenced DNA yield and microbiota results. The DNA isolation kits with bead-beating, the PS and MX kits, gave the closest representation of the expected mock community. These kits also produced the highest milk DNA yield, suggesting that they lysed bacteria more effectively. Importantly, we re-confirmed that a higher milk DNA yield is associated with less contamination [52].

Assessing and minimising contamination is particularly important for low-biomass samples [55]. Our results emphasise this as three of the four high-biomass bacterial mock communities we isolated had no 16S rRNA gene sequencing reads belonging to unexpected bacteria, whereas the low-biomass negative control and milk samples were potentially contaminated. Contaminants likely originated from DNA isolation kits as (1) isolation-negative controls had more contamination than library preparation negative controls, (2) all samples were processed in the same environment and (3) the extent and nature of contamination differed between kits. Indeed, the potentially contaminating bacteria we identified, e.g. *Cutibacterium* (formerly *Propionibacterium*), have previously been suggested as kit contaminants [55]. While there are various approaches for identifying and removing potential contaminants from sequencing data [55], it remains difficult to balance removal of potential contaminant signals against retaining true signals. It is therefore important to choose a DNA isolation method that introduces as little contamination as possible. Milk samples isolated with the PS kit shared only a median of 3% and a maximum of 10% of sequencing reads with corresponding negative controls, suggesting they were less affected by potential contamination than samples isolated with other kits.

### Bacterial enrichment methods did not improve milk metagenomic sequencing data

The HL-PMA and HL-Benz enrichment methods have previously been shown to decrease human metagenomic sequencing read-depth and increase the microbial metagenomic sequencing read-depth in low-biomass saliva (HL-PMA) [26] and sputum samples (HL-Benz) [25]. Both methods rely on hypotonic lysis of human cells and depletion of liberated DNA to enrich bacterial DNA. Unfortunately, when applied to human milk, our enriched samples either failed sequencing or still had ≥98.8% human reads. One previous study that investigated the performance of bacterial enrichment methods in milk, including HL-PMA, described it as yielding insufficient DNA for further analysis [56]. Human milk is rich in nutrients, which potentially interfere with DNA isolation [12], and other human milk components also likely hinder bacterial enrichment. An enrichment method that effectively reduces human host contamination and enables the use of metagenomic sequencing on human milk thus remains to be found.

### Sequencing methods affect microbiota results

As sequencing of 16S-ITS-23S rRNA gene amplicons has enabled tracking of bacterial strains in infant guts [57], we tested whether 16S-ITS-23S rRNA gene sequencing could improve bacterial classification and detect bacterial sharing events in low-biomass human milk samples. Our results from the bacterial mock communities show that, although bacteria were often assigned on the species level, this classification was not always correct. As 16S-ITS-23S rRNA gene sequencing detected more ASV-sharing in related mother-infant pairs compared to unrelated pairs, we suggest that the ~2500 bp-long 16S-ITS-23S rRNA gene amplicons contain sufficient information to provide taxonomic information below genus level. It may be that high variation in the ITS region complicated alignment to reference databases and the databases with 16S-ITS-23S rRNA gene sequences require further curation.

16S-ITS-23S rRNA gene sequencing missed the *Escherichia*, *Limosilactobacillus* and *Pseudomonas* in the bacterial mock community. However, importantly, they were detected in the bacterial mock community with 16S rRNA gene sequencing and in milk and faeces with 16S-ITS-23S rRNA gene sequencing, suggesting that their DNA was isolated from the bacterial mock community but not successfully amplified during 16S-ITS-23S library preparation or that sequencing reads were not assigned their respective taxonomy during bioinformatic processing. The primers chosen, sequencing method and database can all affect microbiota results [58, 59], and they also likely affect 16S-ITS-23S rRNA gene sequencing results. Indeed, compared to 16S rRNA gene sequencing, 16S-ITS-23S rRNA gene sequencing changed the microbiota results of infant oral and maternal faecal samples, although not those of milk and infant faeces. It is possible that the sample size was too small to detect differences between sequencing methods for the latter. On a positive note, 16S-ITS-23S rRNA gene sequencing showed remarkably little contamination. Ultimately, it remains important to assess and report a technique’s limitations and the bias it might introduce.

### 16S-ITS-23S rRNA gene sequencing can detect bacterial transmission

Even though studies based on 16S rRNA gene sequencing report sharing of milk bacteria with other maternal and infant body sites [5, 6, 9, 13, 16, 60–63], 16S rRNA gene sequencing is limited in its ability to study bacterial transmission. Both full-length 16S rRNA gene amplicon sequencing and targeted sequencing of the ITS region between the 16S and 23S rRNA genes improved resolution in previous studies [64, 65]. As expected, the combined 16S-ITS-23S rRNA gene sequencing approach could improve resolution for bacterial transmission studies by detecting more variation in the ~2500 bp-long 16S-ITS-23S rRNA gene amplicons compared to the ~450 bp-long 16S rRNA gene amplicons.

The origin of human milk bacteria is still an area of study. The maternal areolar skin and the infant oral cavity have been suggested to host bacteria that could reach the mammary gland via retrograde transfer [5, 7, 8], while the entero-mammary pathway hypothesis suggests that maternal gut bacteria can travel to the mammary gland and enter human milk [4, 7, 8, 60, 66]. However, studies on human milk bacterial transfer have been complicated by difficulties with obtaining high-resolution milk bacterial profiles. In our study, using 16S-ITS-23S rRNA gene sequencing data, human milk shared *Rothia*, *Streptococcus*, *Gemella*, *Pauljensenia* and *Veillonella* with the infant’s oral cavity, supporting 16S rRNA sequencing-based studies that report sharing of oral bacteria between a mother’s milk and her infant’s oral cavity [16, 61]. Unfortunately, our approach did not allow to study the direction of a potential bacterial transfer. In contrast to small earlier studies supporting the entero-mammary pathway hypothesis [8, 60, 66], for example, by identifying shared *Bifidobacterium* strains in maternal faeces and milk using cultures and (reduced) metagenomic sequencing, we found no bacterial sharing between maternal faeces and milk. Even though 16S-ITS-23S rRNA gene sequencing detected *Bifidobacterium* genera in infant faeces, no *Bifidobacterium* was detected as potentially shared in our maternal faeces-milk pairs. Larger studies with high-resolution data are needed to better define the roles of different maternal and infant body sites in shaping the human milk bacterial community.

Next to human milk sharing bacteria with the infant oral cavity, we also detected sharing of human milk *Streptococcus* and *Staphylococcus* with infant faeces. This finding is in line with other publications suggesting transfer of bacteria between a mother’s milk and her infant’s gut [5, 6, 8–10, 13, 16, 60, 61, 63]. The 16S-ITS-23S rRNA gene sequencing technique reported here could help identify bacterial sharing between low-biomass human milk samples and infant body sites. Still, future studies are needed to further explore the role of human milk-derived bacteria in infant oral and gut bacterial colonisation. For example, the functions of shared bacteria remain unclear and, while a recent study showed that human milk harbours viable and non-viable bacteria [67], their effect on infants remains to be elucidated.

### Limitations and strengths

This study had several limitations. The small sample size limited statistical testing and the ability to draw conclusions. We did not investigate the effect of sample storage on the results, however, we expect only limited effects of (long-term) cold storage on the non-viable microbiota based on previous studies [68–73]. In addition, fat was removed from milk prior to DNA isolation, which likely led to a loss of bacteria within the fat layer [53, 74]. The DNA isolation methods we tested did not distinguish between viable and non-viable bacteria. The 16S-ITS-23S rRNA gene sequencing method detected fewer bacteria compared to the 16S rRNA gene sequencing method, potentially missing (shared) bacteria in the different sample types. A major strength of this study is that we tested various methods on the same samples and applied the same methodology (DNA isolation, sequencing, bioinformatic processing) to the steps not under study. Mock and negative controls added during DNA isolation and sequencing allowed us to identify biases and potential contaminants. We also report how to adapt the PS kit for sample types other than milk. Finally, using long-read 16S-ITS-23S rather than short-read 16S rRNA gene sequencing enabled higher-resolution study of milk bacterial transmission.

## Conclusion

In conclusion, DNA isolation with the PS or MX kit and 16S rRNA gene sequencing reliably profiled the milk microbiota and, though our analysis was limited to 14 mother-infant pairs, long-read 16S-ITS-23S rRNA gene sequencing provided evidence for bacterial transfer from breastfeeding mothers to infants, but not from the maternal gut to breast milk. Our findings pave the way for large milk microbiota studies and 16S-ITS-23S rRNA gene sequencing can help to study bacterial transmission in low-biomass (human milk) samples.

### Supplementary information

Supplementary methods and figures

Supplementary tables

Dataset 1

Dataset 2

Dataset 3

## Data Availability

The sequencing data has been uploaded to the European Genome-phenome Archive (EGAS00001007592). Data files are available as part of the supplementary material. Scripts to process and analyse data can be found on GitHub (https://github.com/GRONINGEN-MICROBIOME-CENTRE/MilkMethods2023).
