# Supplementary material for: Analysis of microbial composition and sharing in low-biomass human milk samples: a comparison of DNA isolation and sequencing techniques
Source: ISME Commun. 2023 Nov 9;3:116. doi: 10.1038/s43705-023-00325-6 (PMC10636111; doi:10.1038/s43705-023-00325-6)
Supplement: Supplementary file 1 — Supplementary methods and figures [file 43705_2023_325_MOESM1_ESM.pdf]

SUPPLEMENTARY MATERIAL

Supplementary methods

Quantitative PCR

BaseClear performed a quantitative PCR (qPCR) assay targeting the 16S rRNA gene to estimate the total bacterial DNA content of samples[1]. Each 10 µl reaction consisted of 5 µl ABsolute™ Blue qPCR Mix (low ROX, AB4318B, Thermo Fisher), 0.2 µl forward primer (5’-CGGTGAATACGTTCYCGG-3’, 10 µM), 0.2 µl reverse primer (5’-GGWTACCTTGTTACGACTT-3’, 10 µM), 0.1 µl probe (FAM-CTTGTACACACCGCCCGTC-BHQ1, 10 µM), 2 µl PCR grade water (AM9935, Thermo Fisher) and 2.5 µl 2-fold diluted DNA. The qPCR assay was run in an Applied Biosystems QuantStudio™ 5 Real-Time PCR system (A34322, Thermo Fisher) using this program: 15 min at 95°C, 40 cycles of 15 s at 95°C and 1 min at 52°C. A standard curve with 1.1 x 10^1 to 1.1 x 10^8 16S rRNA gene copies per 1 µl was prepared by eight 10-fold serial dilutions of a 192-bp long synthesised, cloned, linearised and purified 16S rRNA gene DNA product (CTAGTAATCGTGGATCAGAATGCCACGGTGAATACGTTCCCGGGCCTTGTACAC ACCGCCCGTCACACCATGGGAGTGGGTTGCAAAAGAAGTAGGTAGCTTAACCTT CGGGAGGGCGCTTACCACTTTGTGATTCATGACTGGGGTGAAGTCGTAACAAGG TAACCGTAGGGGAACCTGCGGTTGGATCAC, produced by Genscript, Rijswijk, Netherlands). The slope of the standard curve was -3.52, the y-intercept was 38.8, the efficiency was 92.3% and R^2 was 0.998. All samples, standards and no template controls were processed in triplicates in a single qPCR assay. The triplicates for the no template control had Cq values of 36.0, 36.9 and >40, while PS kit-DNA isolation negative controls had Cq values ≥36.4. The coefficient of variation for sample replicates was ≤1.2%. Results were normalised per 1 ml human milk sample input for DNA isolation or per 1 ng DNA used as input for the qPCR assay.

Bacterial enrichment

We tested two bacterial enrichment methods on the method test samples. Both hypotonically lysed human cells and degraded freed DNA prior to DNA isolation. Milk and negative control samples were prepared in triplicate with each enrichment method, while the bacterial mock community was prepared once with each method.

Hypotonic lysis and DNA degradation with benzonase endonuclease

The hypotonic lysis and benzonase treatment (HL-Benz) method was adapted from Nelson et al. 2019[2], who successfully used it for reducing human host contamination in complex respiratory samples prior to DNA isolation and metagenomic sequencing.

For hypotonic lysis, 5.5 ml filtered DEPC-treated water was added to 3.5 ml milk and negative controls, respectively. For the positive control, 8.9 ml filtered DEPC-treated water were added to 75 µl bacterial mock community sample. Samples were incubated with gentle agitation at room temperature (20 to 25°C) for 1 h.

To deplete freed DNA, 1 ml 10X benzonase buffer (200 mM Tris-HCl pH 8.0 (15568-025, Invitrogen), 10 mM $MgCl_2$ (M8266, Sigma-Aldrich, St. Louis, Missouri, USA)), and 1 µl benzonase (250 U, E-1014, Sigma-Aldrich) were added to each sample and incubated with gentle agitation at 37°C for 2 h.

Samples were centrifuged at 8 000 x *g* for 10 min to pellet bacterial cells, and fat and supernatants were removed. Bacterial pellets were resuspended in 1 ml phosphate-buffered saline (PBS, P5119, Sigma-Aldrich), moved to 2-ml tubes, centrifuged at 8 000 x *g* for 10 min, and supernatants were removed. Pellets were then resuspended in 1 ml Tris-EDTA, pH 8.0, (TE, 93283, Sigma-Aldrich) and 10 µl of 0.5 M EDTA, pH 8.0, (15575-038, Invitrogen) were added to inactivate the benzonase. Afterwards, samples were again centrifuged at 8 000 x *g* for 10 min and bacterial pellets were washed by addition of 1 ml PBS, centrifugation at 8 000 x *g* for 10 min, and removal of supernatants. Finally, bacterial pellets were resuspended in 175 µl PBS and stored at -20°C until DNA extraction.

Hypotonic lysis and DNA degradation with propidium monoazide and light

The hypotonic lysis and propidium monoazide treatment (HL-PMA) method was adapted from Marotz et al.[3], who successfully used it to remove human host cell DNA from saliva samples prior to metagenomic sequencing.

For hypotonic lysis, 10.5 ml filtered DEPC-treated water was added to 3.5 ml milk and negative controls, respectively. For the positive control, 13.9 ml filtered DEPC-treated water were added to 75 µl bacterial mock community sample. Samples were incubated with gentle agitation at room temperature for 1h.

Samples were centrifuged at 9 400 x *g* for 10 min, and fat and supernatants were removed. Pellets were resuspended in 175 µl filtered DEPC-treated water and moved to 2-ml tubes. To inactivate freed DNA, 1.75 µl of 1 mM propidium monoazide (PMA, 40013, Biotium, Fremont, California, USA) were added, samples were briefly vortexed, and incubated in the

dark at room temperature for 5 min. Then, the sample tubes were placed horizontally on ice 15 cm below a blue lamp (Paulmann 128970 PMQ 11/18 230V BLUE 1W, Paulmann, Springe-Völksen, Germany) and incubated for 50 min with gentle agitation. Samples were stored at -20°C until DNA extraction.

DNA isolation and quantification

After bacterial enrichment, samples were used for DNA isolation with the PS kit and Qubit-based DNA quantification as described in the Methods section. DNA eluates were stored at -20ºC until further processing.

Shotgun metagenomic sequencing and data processing

DNA was sent to the Institute of Clinical Molecular Biology at the Christian-Albrecht University of Kiel in Germany for shotgun metagenomic sequencing. Sequencing libraries were prepared using the NEBNext Ultra II DNA Library Prep Kit for Illumina (E7645L, NEB, Ipswich, Massachusetts, USA) and sequenced on a NovaSeq 6000 (2x 150 bp, Illumina).

Shotgun metagenomic sequencing data was processed using an approach previously described for the Lifelines Dutch Microbiome Project[4]. In brief, the kneaddata pipeline (version 0.10.0) from bioBakery 3[ref.5] was used to trim raw sequencing reads to PHRED score 30, to remove sequencing adapters, and to remove reads aligning to the human genome (GRCh37/hg19). Taxonomic composition of samples was profiled using MetaPhlAn4 (version 4.0.1) using the marker gene database ChocoPhlAn (version vJan21)[5].

References for the supplementary material

1 Suzuki MT, Taylor LT, DeLong EF. Quantitative analysis of small-subunit rRNA genes in mixed microbial populations via 5'-nuclease assays. *Appl Environ Microbiol* 2000; **66**: 4605–4614.

2 Nelson MT, Pope CE, Marsh RL, Wolter DJ, Weiss EJ, Hager KR *et al.* Human and extracellular DNA depletion for metagenomic analysis of complex clinical infection samples yields optimized viable microbiome profiles. *Cell Rep* 2019; **26**: 2227–2240.

3 Marotz CA, Sanders JG, Zuniga C, Zaramela LS, Knight R, Zengler K. Improving saliva shotgun metagenomics by chemical host DNA depletion. *Microbiome* 2018; **6**: 42.

4 Gacesa R, Kurilshikov A, Vich Vila A, Sinha T, Klaassen MAY, Bolte LA *et al.* Environmental factors shaping the gut microbiome in a Dutch population. *Nature* 2022; **604**: 732–739.

5 Beghini F, McIver LJ, Blanco-Míguez A, Dubois L, Asnicar F, Maharjan S *et al.* Integrating taxonomic, functional, and strain-level profiling of diverse microbial communities with bioBakery 3. *Elife* 2021; **10**: e65088.

Supplementary figures

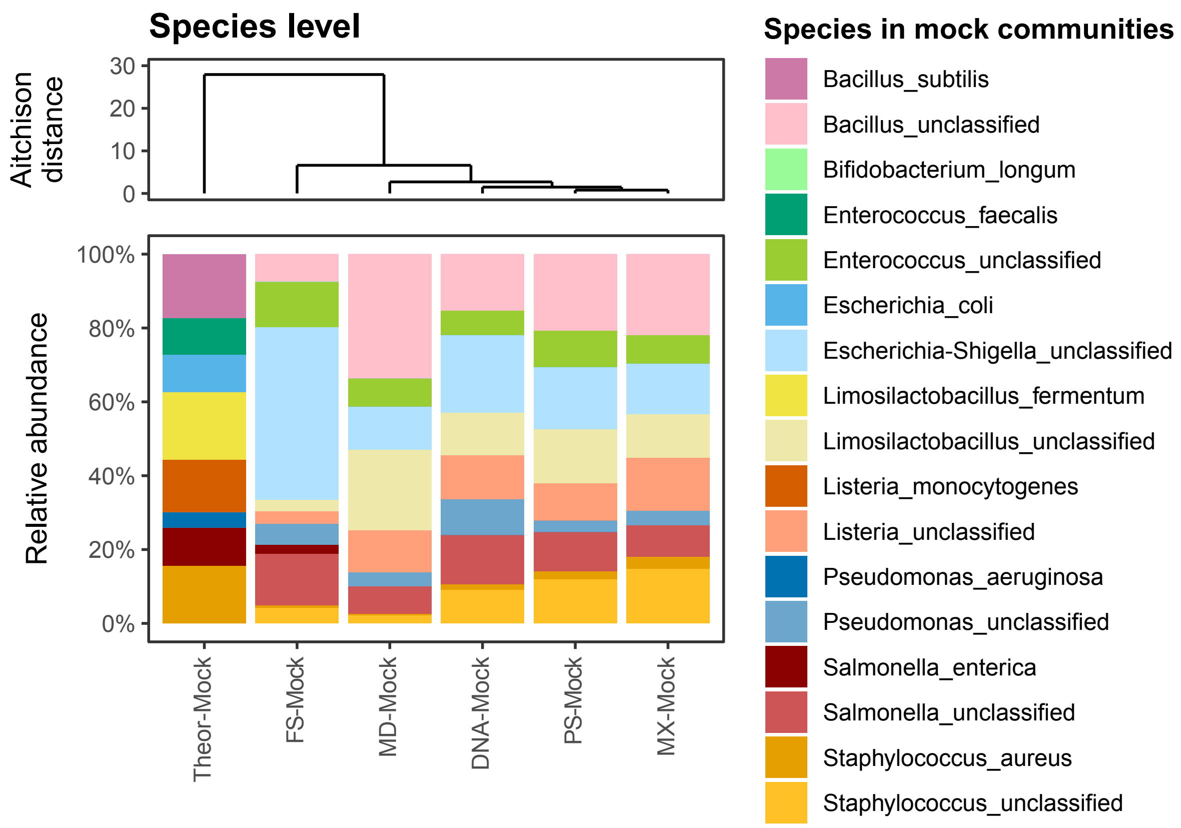

Figure S1: Mock community composition by DNA isolation method.

DNA was isolated from bacterial mock communities using four DNA isolation kits, and samples were sequenced using 16S rRNA gene sequencing. Bacterial taxonomies were assigned using the SILVA database. The plot shows the relative abundances of bacterial species in mock communities, including bacterial mock communities isolated with the four DNA isolation kits, a DNA mock community (DNA-Mock, added at the start of library preparation) and a theoretical mock community (Theor-Mock, see Table S1). Mock communities are clustered based on Aitchison distances. FS = QIAamp Fast DNA Stool Mini kit, MD = Milk Bacterial DNA Isolation kit, PS = DNeasy PowerSoil Pro kit, MX = MagMAX Total Nucleic Acid Isolation kit.

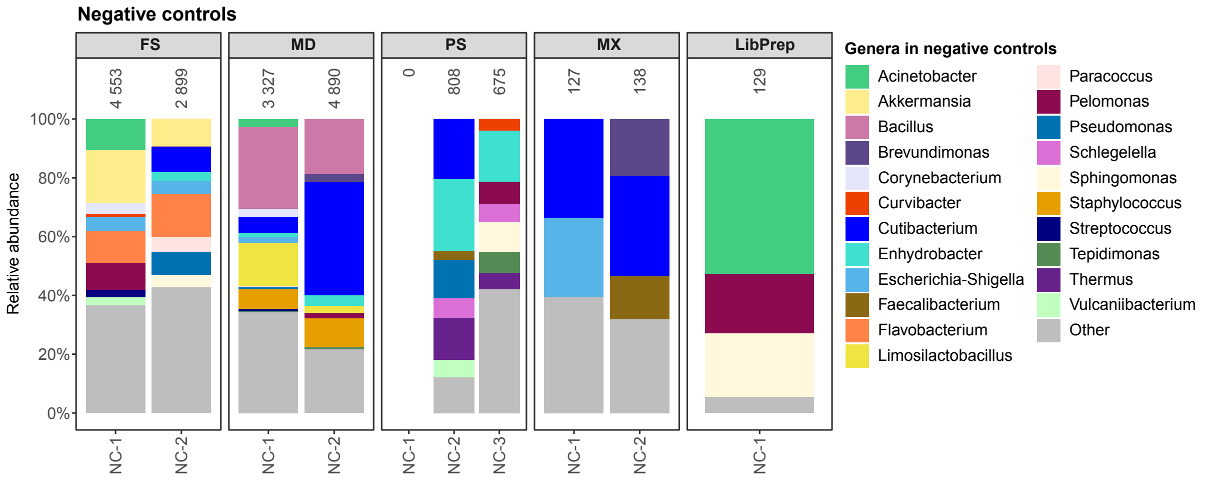

Figure S2: Bacterial genera in negative controls by DNA isolation method.

DNA was isolated from filtered DEPC-treated water using four DNA isolation kits and samples were sequenced using 16S rRNA gene sequencing. Bacterial taxonomies were assigned using the SILVA database. The plot shows the relative abundances of bacterial genera. Only classified bacterial genera present in at least two samples with at least 2% relative abundance in any sample are shown. Genera present at lower prevalence and abundance or without genus-level classification are summarised in 'Other'. Numbers above the bars indicate the number of sequencing reads of the respective sample. NC = Negative control, FS = QIAamp Fast DNA Stool Mini kit, MD = Milk Bacterial DNA Isolation kit, PS = DNeasy PowerSoil Pro kit, MX = MagMAX Total Nucleic Acid Isolation kit.

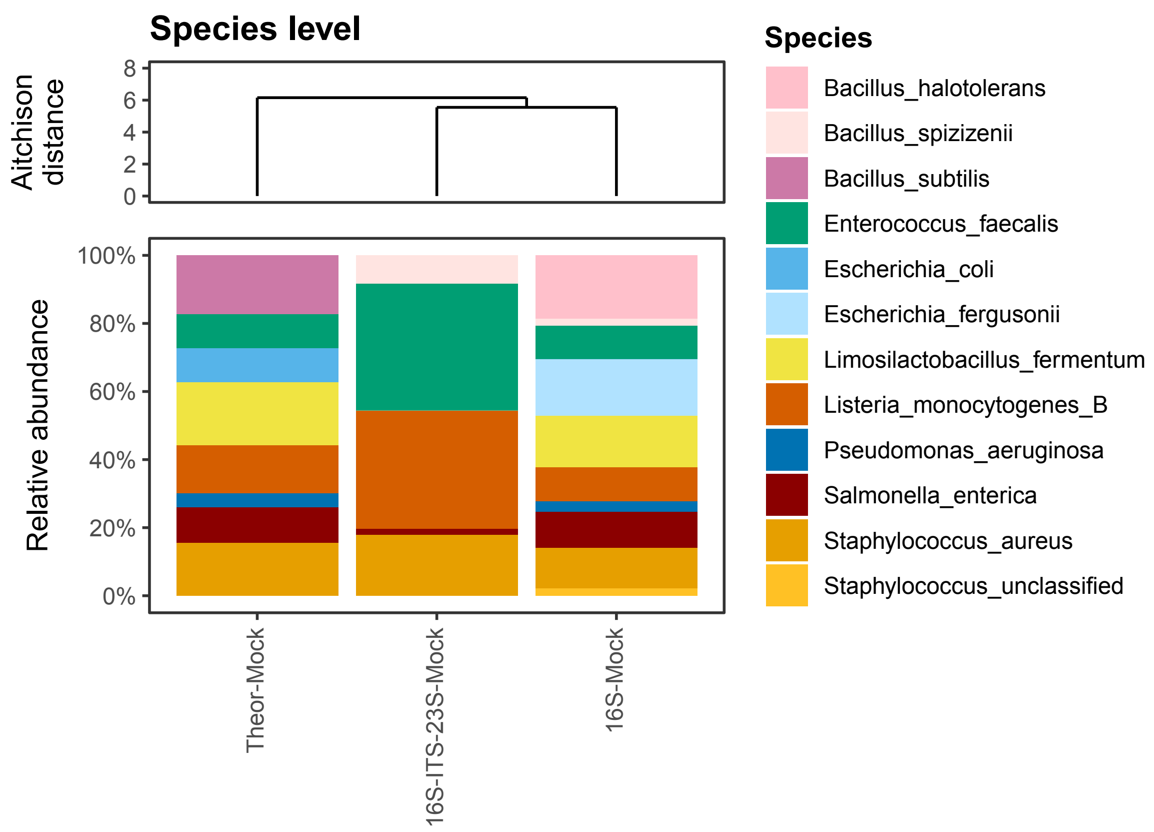

Figure S3: Mock community composition by sequencing method.

DNA was isolated from bacterial mock communities using the DNeasy PowerSoil Pro kit, and samples were sequenced using 16S and 16S-ITS-23S rRNA gene sequencing. Bacterial taxonomies were assigned using the FANGORN database. The plot shows the relative abundances of bacterial species in the sequenced mock communities and a theoretical mock community (Theor-Mock, see Table S1). Mock communities are clustered based on Aitchison distances.

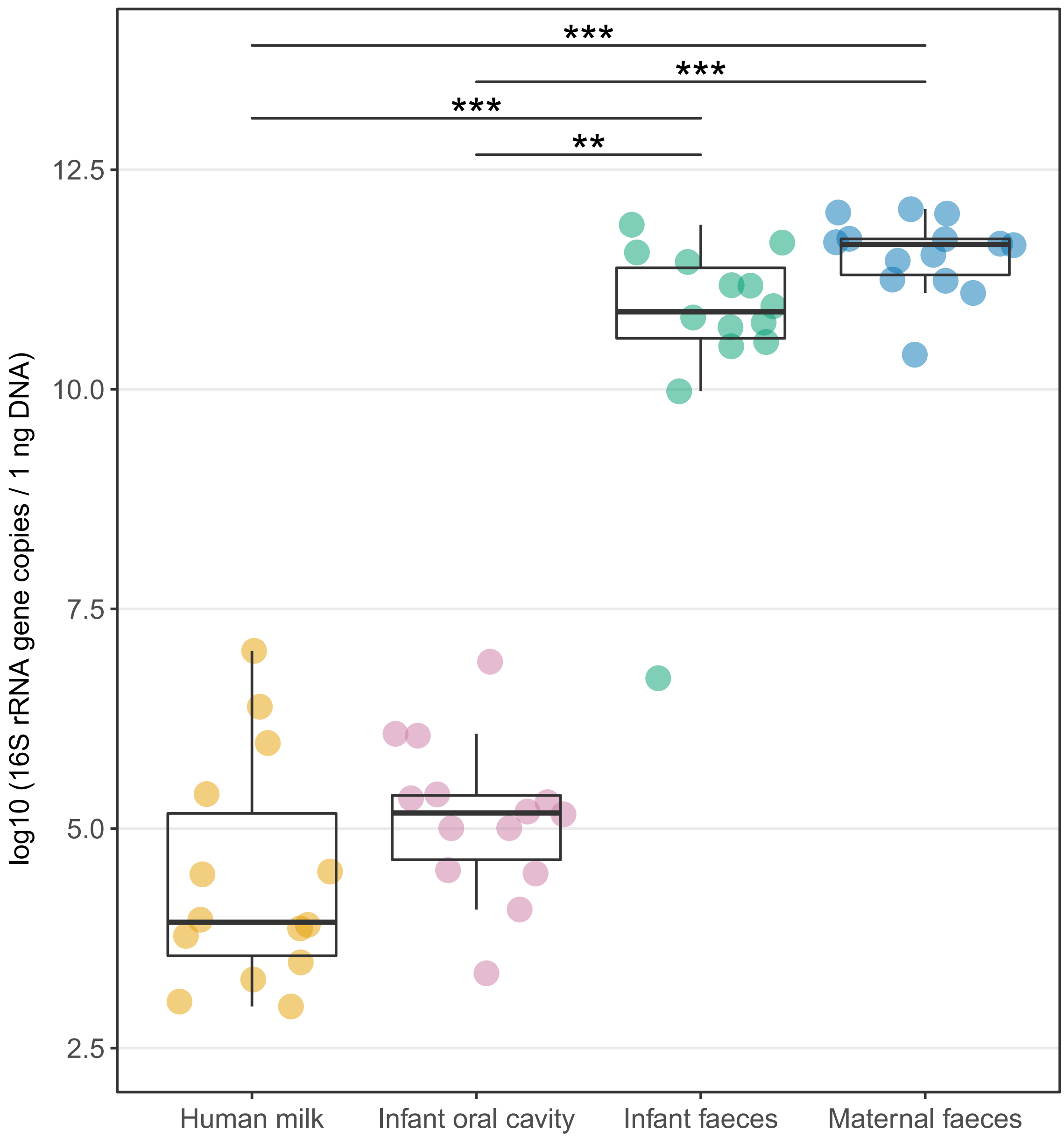

Figure S4: Total bacterial load by sample type.

DNA was isolated from human milk, infant oral swabs, infant faeces, and maternal faeces from 14 Lifelines NEXT mother-infant pairs using the DNeasy PowerSoil Pro kit. Total bacterial load (16S rRNA gene copy numbers) was assessed using quantitative PCR (qPCR), normalised to 1 ng sample DNA and log-transformed for plotting. The boxplots show medians with 25% and 75% percentiles and 1.5x the interquartile range. The total bacterial load differed significantly between sample types (Kruskal-Wallis test with Dunn's posthoc test). Statistical significance: **FDR<0.01, ***FDR<0.001. Note that sample input for DNA isolation differed between sample types, *i.e.*, human milk DNA was isolated from 3.5 ml human milk, maternal

and infant faecal DNA was isolated from 0.2–0.5 g faeces, and infant oral DNA was isolated from one cotton swab (see Methods).

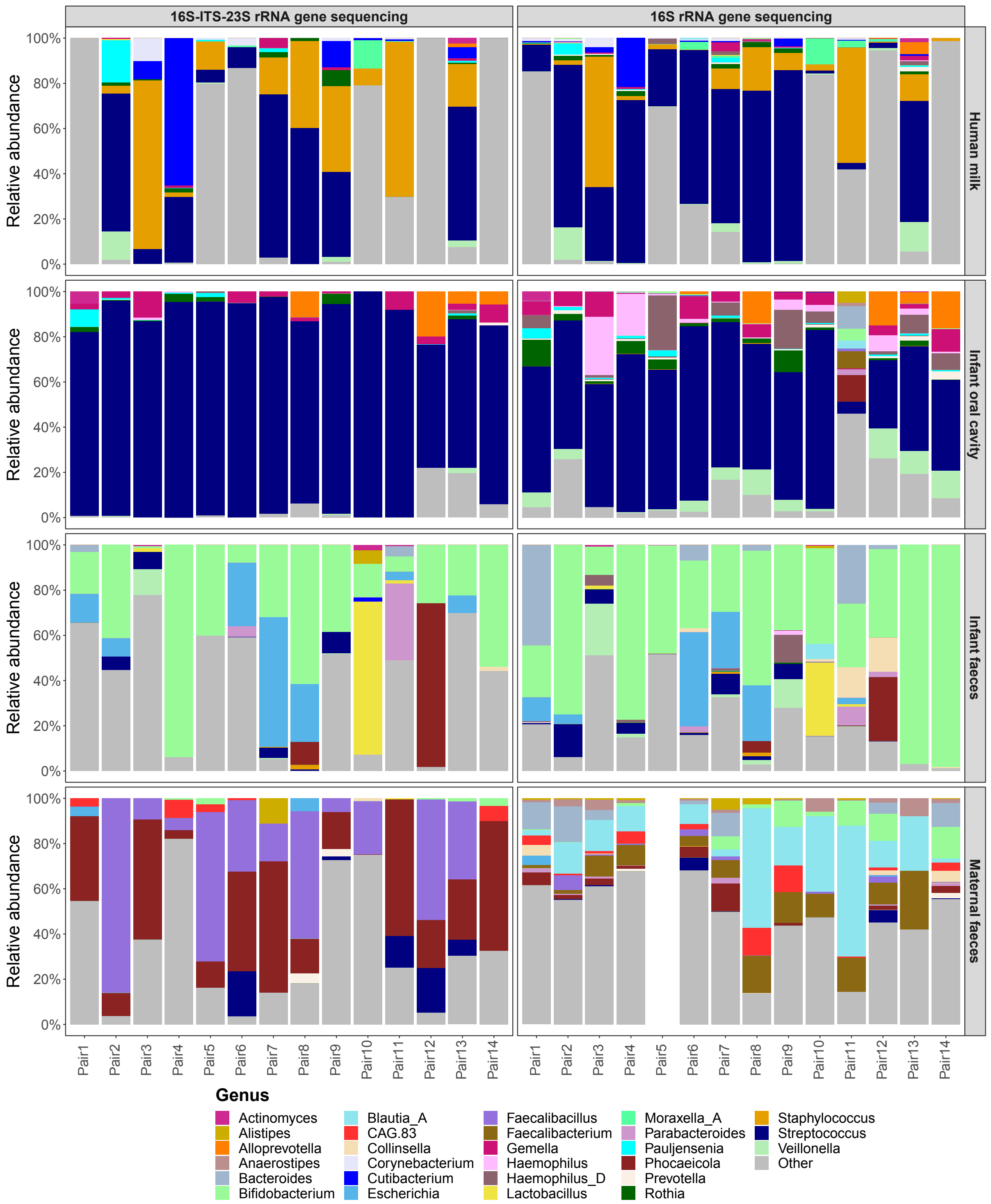

Figure S5: Bacterial composition by sequencing method and sample type.

DNA was isolated from human milk, infant oral swabs, infant faeces, and maternal faeces from 14 Lifelines NEXT mother-infant pairs using the DNeasy PowerSoil Pro kit. Samples were sequenced using 16S and 16S-ITS-23S rRNA gene sequencing and bacterial taxonomies were assigned using the FANGORN database. The plot shows the relative abundances of classified bacterial genera present in at least 14 samples with at least 2% relative abundance in any

sample. Genera present at lower prevalence and abundance or without genus-level classification are summarised in ‘Other’. The maternal faecal sample from pair 5 failed 16S rRNA gene sequencing.

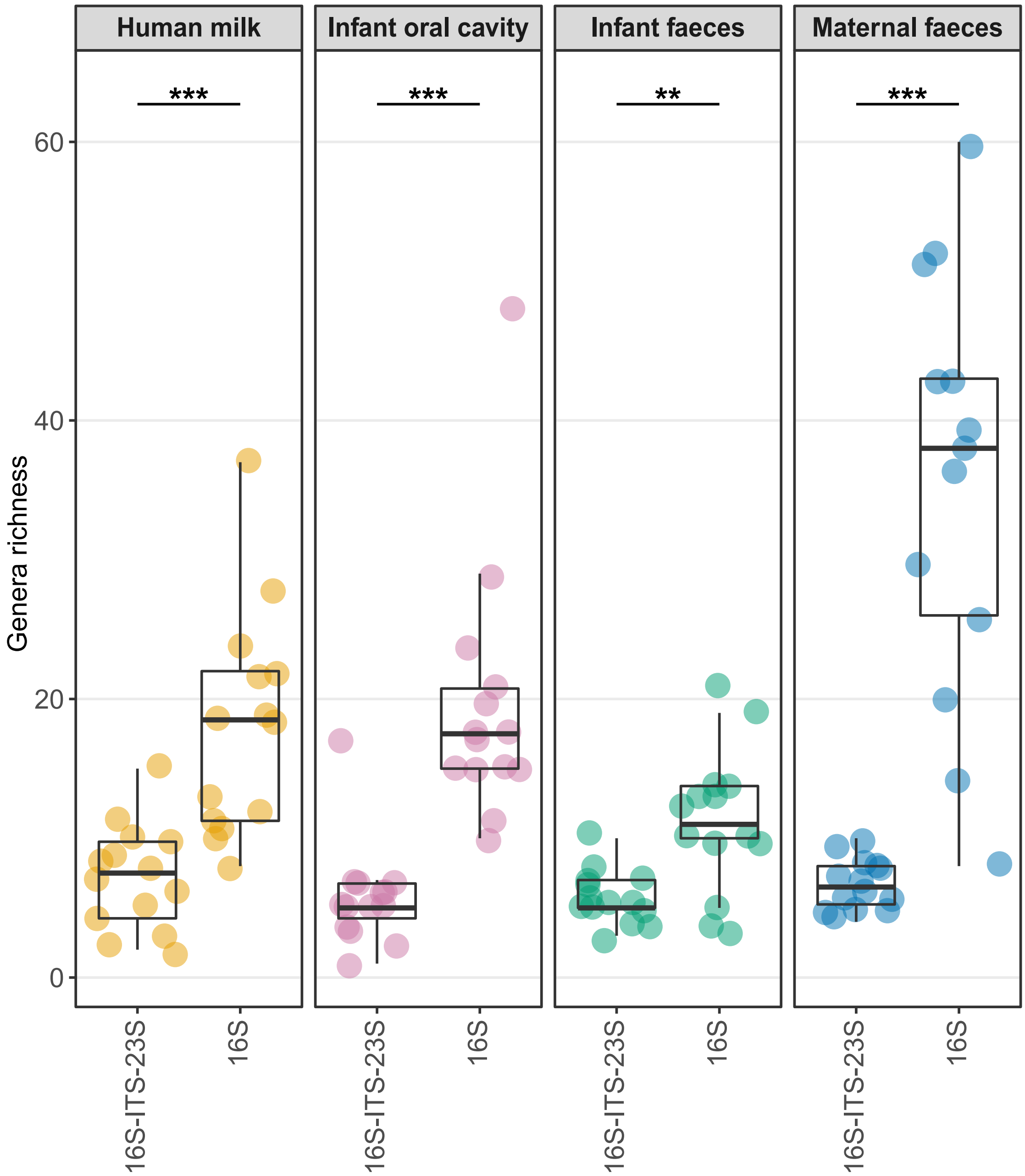

Figure S6: Genera richness by sequencing method and sample type.

DNA was isolated from human milk, infant oral swabs, infant faeces, and maternal faeces from 14 Lifelines NEXT mother-infant pairs using the DNeasy PowerSoil Pro kit. Samples were sequenced using 16S and 16S-ITS-23S rRNA gene sequencing and bacterial taxonomies were assigned using the FANGORN database. The plot shows the number of classified bacterial genera (genera richness) for each sample type and sequencing method. The boxplots represent medians with 25% and 75% percentiles and 1.5x the interquartile range. One maternal faecal sample failed 16S rRNA gene sequencing. Statistically significant results (Mann-Whitney *U* tests): **FDR<0.01, ***FDR<0.001.

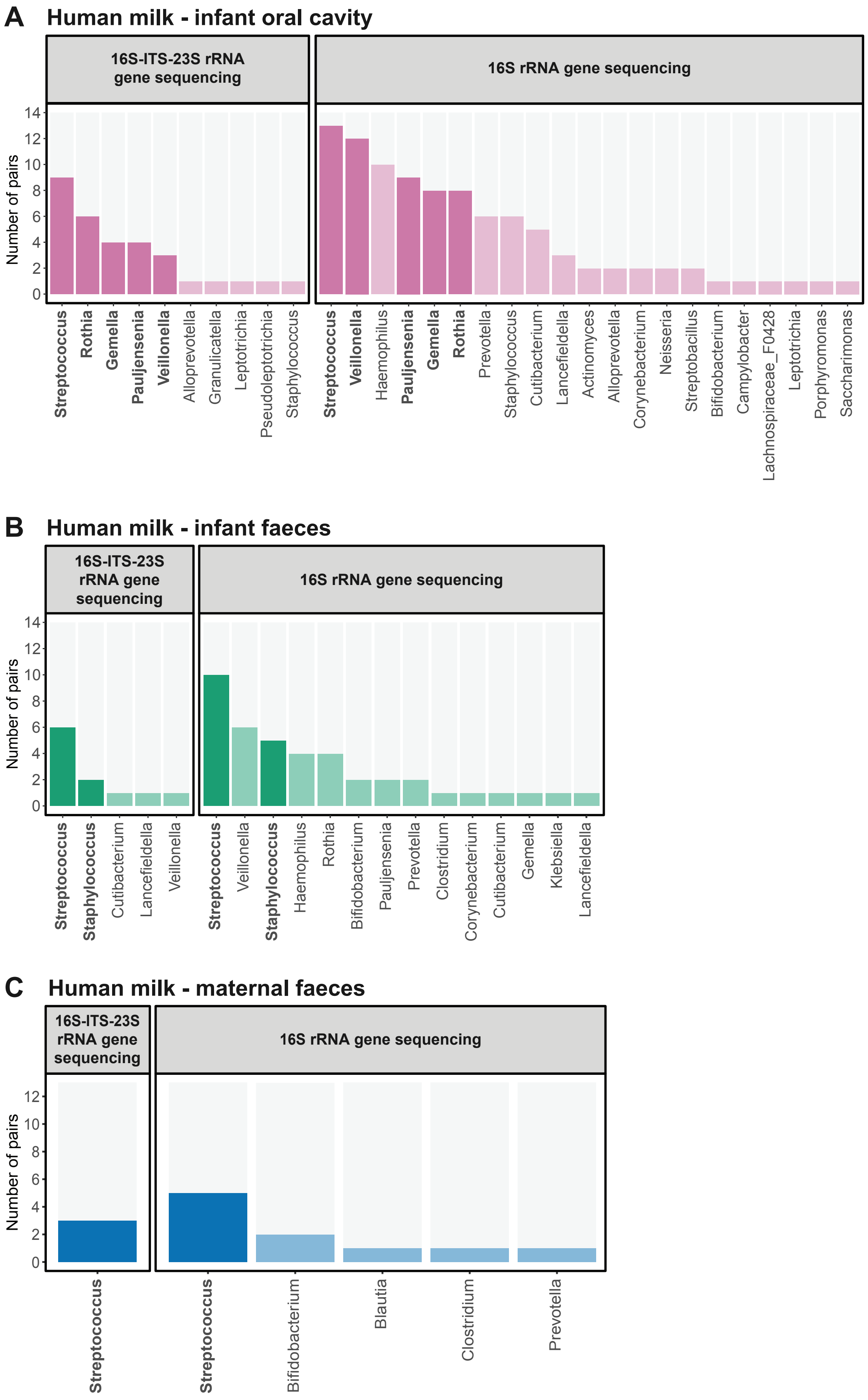
A Human milk - infant oral cavity
16S-ITS-23S rRNA gene sequencing
16S rRNA gene sequencing
Number of pairs
0
2
4
6
8
10
12
14
Streptococcus
Rothia
Gemella
Pauljensenia
Veillonella
Alloprevotella
Granulicatella
Leptotrichia
Pseudoleptotrichia
Staphylococcus
Streptococcus
Veillonella
Haemophilus
Pauljensenia
Gemella
Rothia
Prevotella
Staphylococcus
Cutibacterium
Lancefieldella
Actinomyces
Alloprevotella
Corynebacterium
Neisseria
Streptobacillus
Bifidobacterium
Campylobacter
Lachnospiraceae_F0428
Leptotrichia
Porphyromonas
Saccharimonas
B Human milk - infant faeces
16S-ITS-23S rRNA gene sequencing
16S rRNA gene sequencing
Number of pairs
0
2
4
6
8
10
12
14
Streptococcus
Staphylococcus
Cutibacterium
Lancefieldella
Veillonella
Streptococcus
Veillonella
Staphylococcus
Haemophilus
Rothia
Bifidobacterium
Pauljensenia
Prevotella
Clostridium
Corynebacterium
Cutibacterium
Gemella
Klebsiella
Lancefieldella
C Human milk - maternal faeces
16S-ITS-23S rRNA gene sequencing
16S rRNA gene sequencing
Number of pairs
0
2
4
6
8
10
12
Streptococcus
Streptococcus
Bifidobacterium
Blautia
Clostridium
Prevotella

Figure S7: Bacterial genera detected in human milk and other maternal and infant sample types.

DNA was isolated from human milk, infant oral swabs, infant faeces, and maternal faeces from 14 Lifelines NEXT mother-infant pairs using the DNeasy PowerSoil Pro kit. Samples were sequenced using 16S and 16S-ITS-23S rRNA gene sequencing and bacterial taxonomies were assigned using the FANGORN database. One maternal faecal sample failed 16S rRNA gene sequencing. The bars show the number of pairs, which have the indicated genus detected both in milk and **(A)** the infant oral cavity, **(B)** infant faeces, and **(C)** maternal faeces. Only genera shared in at least one sample-pair are shown. The genera detected in at least two pairs with both sequencing methods (highlighted in bold and with darker colour) were investigated more closely for bacterial sharing based on exact ASV matching (see Figure 3C).

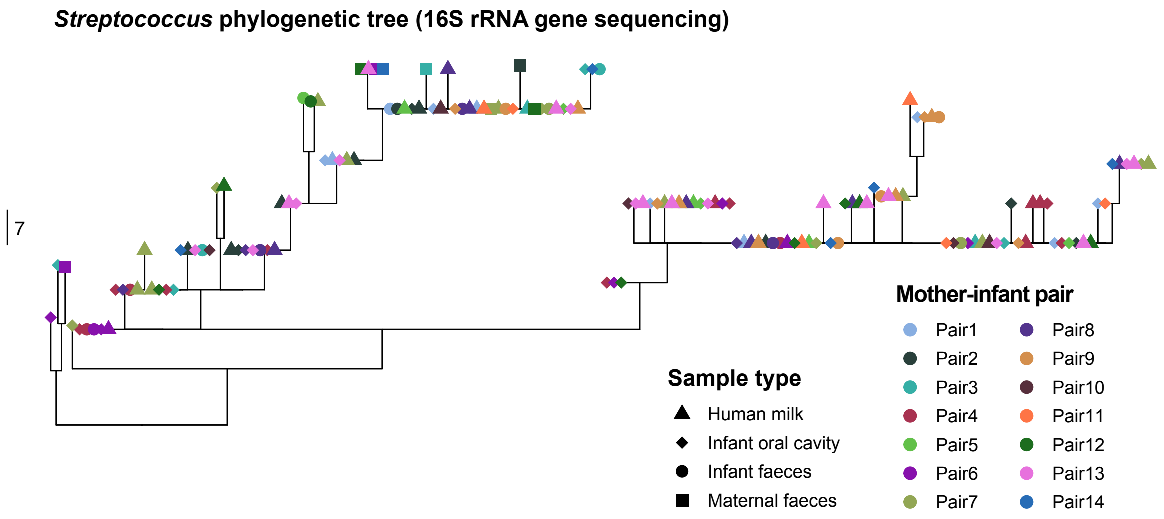

Figure S8: *Streptococcus* phylogenetic tree based on 16S rRNA gene sequencing data. DNA was isolated from human milk, infant oral swabs, infant faeces, and maternal faeces from 14 Lifelines NEXT mother-infant pairs using the DNeasy PowerSoil Pro kit. Samples were sequenced using 16S rRNA gene sequencing and bacterial taxonomies were assigned using the FANGORN database. The maternal faecal sample from pair 5 failed 16S rRNA gene sequencing. The plot shows a phylogenetic tree for *Streptococcus* based on 16S rRNA gene sequencing data. Dot shape indicates sample type. Dot colour indicates the mother-infant pair ID. Phylogenetic distances were log-transformed for plotting.
